# Anti-thymocyte globulin could improve the outcome of allogeneic hematopoietic stem cell transplantation in patients with highly aggressive T-cell tumors

**DOI:** 10.1038/bcj.2015.54

**Published:** 2015-07-31

**Authors:** J Yang, Y Cai, J L Jiang, L P Wan, S K Yan, C Wang

**Affiliations:** 1 Department of Hematology, Shanghai General Hospital, Shanghai, Jiao Tong University School of Medicine, Shanghai, China

## Abstract

The early experiment result in our hospital showed that anti-thymocyte globulin (ATG) inhibited the proliferation of lymphoid tumor cells in the T-cell tumors. We used the ATG as the part of the conditioning regimen and to evaluate the long-term anti-leukemia effect, the safety and complication in the patients with highly aggressive T-cell lymphomas. Twenty-three patients were enrolled into this study. At the time of transplant, six patients reached first or subsequent complete response, three patients had a partial remission and 14 patients had relapsed or primary refractory disease. The conditioning regimen consisted of ATG, total body irradiation, toposide and cyclophosphamide. The complete remission rate after transplant was 95.7%. At a median follow-up time of 25 months, 16 (69.6%) patients are alive and free from diseases, including nine patients in refractory and progressive disease. Seven patients died after transplant, five from relapse and two from treatment-related complications. The incidence of grades II–IV acute graft-vs-host disease (GvHD) was 39.1%. The maximum cumulative incidence of chronic GvHD was 30%. The most frequent and severe conditioning-related toxicities observed in 8 out of 23 patients were grades III/IV infections during cytopenia. Thus, ATG-based conditioning is a feasible and effective alternative for patients with highly aggressive T-cell tumors.

## Introduction

Aggressive T-cell lymphomas represent 10–15% of non-Hodgkin's lymphomas in adults. Aggressive T-cell lymphomas show a worse prognosis than B-cell lymphoma and T-lineage acute lymphoblast leukemia (ALL) also show a worse prognosis. The probability of long-term disease-free survival (DFS) is <30%.^1^ Patients with these relapsed or refractory diseases are generally considered incurable with conventional therapies. Myeloablative conditioning therapy followed by allogeneic hematopoietic stem cell transplantation (HSCT) may be the good choice for these high-risk patients. Although more intensified conditioning regimens improve the rate of complete remission (CR), the relapse has remained a significant cause of death in the high-risk patients.^2^ Allo-HSCT may be used as the initial treatment in the kind of patients because of such poor prognosis. So the optimal type and timing of transplantation remain to be defined.

A combination of cyclophosphamide (120 mg/kg) and total body irradiation (12 Gy in six fractions) have been used as a standard myeloablative conditioning regimen in ALL patients and aggressive T-cell lymphomass for the past 30 years.^3^ Anti-thymocyte globulin (ATG) is used in allo-HSCT for the prophylaxis and treatment of acute graft-versus-host disease (aGvHD). Its immunosuppressive action is thought to be mainly mediated by T-lymphocyte depletion. The *in vitro* studies have confirmed recently that the ATG also have the killing effect on the tumor cells of the lymphatic system.^4, 5^ Furthermore, its anti-leukemic activity of ATG may be an additive factor to conditioning chemotherapy for cleaning out T-cell tumors and decreasing tumor recurrences without increasing cytotoxicity of hematopoietic cells.

On the basis of these promising results, we conducted a prospective clinical study to observe the safety and efficacy of a conditioning regimen consisting of ATG as well as 10 Gy total body irradiation, cyclophosphamide and etoposide for the patients with high-risk, primary refractory or relapsed T-cell tumors.

## Patients and methods

### Patients

Between April 2006 and May 2015, a total of 23 consecutive cases (male 13, female 10) were enrolled into this study (Table 1). The pathology diagnoses were T-cell acute leukemia (white blood cell count at diagnosis were all above 100 × 10^9^/l), peripheral T-cell lymphoma not otherwise specified (PTCL NOS), hepatosplenic T-cell lymphoma, γ/δT-cell lymphoma, angioimmunoblastic T-cell lymphoma (AITL) and T-lymphoblastoid cell lymphoma (T-LBL). All lymphoma patients had advanced diseases, including 10 patients having bone marrow involvement at diagnosis and 12 patients with B symptoms. A mediastinal mass was present in nine patients with 66.7% cases having a pleural and/or pericardial effusion. Median patient age at the time of transplant was 26 years (range, 7–55 years of age). Before enrollment, written informed consent was obtained from each patient or the patient's legal guardian. The study was registered at clinicaltrials.gov (No. NCT02290132), and it was approved by the Hospital Ethics Committee.

All patients received at least one line of chemotherapy before allo-HSCT. The median course number of prior therapies was 8 (rang, 2–18). All patients received a variety of induction or re-induction regimens before the transplants. Seven patients with aggressive PTCLs were treated with 4–18 courses of chemotherapy and all in PD before transplants. Two patients had undergone a prior autologous HSCT. Eleven patients with T-LBL were treated with 4–14 courses of chemotherapy. Only three patients reached the first completed remission (CR1) before the transplants. The other eight patients failed to response to the second line of salvage therapy. Five patients with T-ALL were treated with 2–16 courses of chemotherapy, including one case in CR1, two cases in CR2 and other two cases in non-remission after the second or third relapses before transplants. At the time of transplantation, a total of six patients reached first or subsequent complete response (CR) with conventional therapy or the salvage therapy, three patients had a partial remission (PR), 11 patients had relapsed disease without responding to salvage therapy or progressive disease and three patients had a primary refractory disease. The median time between diagnosis and transplant was 9 months (range, 3–62 months).

### Clinical evaluation

Based on the criteria of the NCCN guidelines, CR was defined as the disappearance of all clinical, biological and radiological disorders related to lymphoma. Partial response (PR) was defined as at least 50% reduction of the tumor burden. Progressive disease (PD) was defined as more than 25% increase of the tumor mass. The CR criteria for acute leukemia was defined as the presence of a morphologically normal bone marrow and at least 1.5 × 10^9^ granulocytes/l and 100 × 10^9^ platelets/l in the blood. Relapse was defined as >5% leukemic cells in bone marrow aspirates or new extramedullary leukemia in patients with a previously documented CR.

### Transplant conditioning

The conditioning regimen in this study consisted of Rabbit ATG (2.5 mg/kg of body weight (BW) on days −5, −4, −3 and −2 (total dose: 10 mg/kg BW), total body irradiation (10 Gy in five fractions on days −3, −2 and −1), cyclophosphamide (60 mg/kg BW on days −5 and −4 (total dose: 120 mg/kg BW ) and etoposide or teniposide (15 mg/kg BW on days −7 and −6 (total dose: 30 mg/kg BW ) in 19 patients. The one case who had experienced with autologous transplantation received a regimen of ATG, total body irradiation and teniposide. Two patients in CR1 and one patient who was 55 years old underwent a regimen of ATG, total body irradiation and cyclophosphamide.

### Sources of donors

Donor-recipient human leukocyte antigen (HLA) matching was considered for HLA-A, -B, -C, DR and DQ. Donors were 10/10 HLA-matched related (5), 10/10 matched unrelated (5), 8/10 matched unrelated (5), mismatched related (8). Stem cell sources were all from mobilized peripheral blood. The median number of nucleated cells and CD34+ cells in the allografts was 12.28 × 10^8^/kg (range, 6.44–24.85 × 10^8^/kg) and 8.33 × 10^6^/kg (range,1.97–24.85 × 10^6^/kg), respectively.

### Graft-versus-host disease prophylaxis

Graft-versus-host disease (GvHD) prophylaxis was cyclosporine in combination with short term methotrexate. Cyclosporine was dosed i.v. starting on day-7 to achieve a target trough level of 200–300 ng/ml. Immunosuppression agents were adjusted according to quantitative and serial monitoring of T-cell chimerism post transplant.^6^ In the absence of GvHD, immunosuppression was discontinued by 6–9 months after transplant.

### Supportive care

All patients received prophylactic levofloxacin, acyclovir and micafungin from the beginning of conditioning therapy until hematological reconstitution. Prophylactic micafungin (50 mg per day) was administered from the day of the conditioning until 1 month after transplant.^7^ Quantitative real-time PCR assays for CMV DNA were also administered for surveillance in order to determine administration of antivirus medicines in all patients. Preemptive therapy with ganciclovir (5 mg/kg bid) started if CMV DNA was more than 100 copies/ml. Continue induction dosing was needed until viracemia disappearance. Patients were treated with maintenance dose (2.5 mg per day) for 1 week.

### Chimerism

Quantitative chimerism analyses were performed using short-tandem-repeat-based PCR techniques at regular intervals for every 4 weeks after transplant at first 6 months.^6^

### GvHD grading and treatment

Acute GvHD (aGvHD) and chronic GvHD was graded according to the international procedure: grades 0, I, II, III or IV or absent, limited or extensive; respectively.^8^ Initial treatment of aGvHD consisted of prednisone (1–2 mg/kg per day, taper started after 14 days). Intravenous Tacrolimus was resumed at full dosage (0.01 mg/kg per day) and achieved a target trough level of 10–15 ng/ml. Steroid-refractory aGvHD was treated with additional CD25 monoclonal antibodies such as basiliximab.

### Statistical methods

Treatment response, GvHD-onset and overall survival (OS) were calculated from the day of transplant to respective event. Death of what cause ever was counted as an event in case of OS. OS and DFS were measured from the date of transplants and they were estimated according to the Kaplan–Meier method. The statistical analyzes were performed using IBM SPSS 17.0 statistical software (IBM, North Harbour, Portsmouth, UK). Non-relapse mortality was defined as death resulting from any cause related to allo-HSCT and not resulting from disease relapse or progression.

## Result

### Engraftment and chimerism

Neutrophil engraftment was defined as the first of three consecutive days of count >0.5 × 10^9^/l. Platelet engraftment was defined as the first of seven consecutive days with platelet counts of >20 × 10^9^/l. All patients were successfully engrafted. The time needed for neutrophil engraftment varied between 10 and 14 days (median 11 days), whereas platelet counts >20 × 10^9^/l were observed between 11 and 23 days (median 14 days). A complete donor chimerism was observed in all patients on day 28 after HSCT.

### Survival and disease response

The median follow-up time for these patients were 25 months(range, 2–101 months). The disease assessment after allo-HSCT showed that six cases of T-ALL or T-LBL in CR before transplant remained in CR and five patients had long survival. Three cases in PR achieved CR after transplant and two of them died from relapse. From the 14 cases characterized as refractory and progressive disease before transplant, 13 cases achieved CR after allogeneic HSCT. One patient (UPN 14) with primary refractory T-LBL achieved PR on day 30 and soon died from progressive disease. The overall CR rate after transplant was 22/23 (95.7%). The CR rate in the patients without response to common chemotherapy was 13/14 (92.9% Table 2).

There were 16 patients who suffered from T-ALL and T-LBL according to the histopathological subtypes and had a stage III or IV disease at the time of diagnosis. Among other 10 cases of PR or PD at the time of transplant, nine cases reached CR post transplant. Three of them died within 100 days post transplant. Two of them died from severe aGvHD. One patient who could not achieve CR after alloSCT died of disease progression on month 3.

Six cases of PTCL and one case of angioimmunoblastic T-cell lymphoma had advanced stages at the transplant and they remained alive without evidence of diseases. The patient who had undergone a prior autologous HSCT was the longest survivor for 86 months. The other six patients had survived for at least 12 months.

Among the twenty-two CR cases, four cases (UPN 7, 10, 15 and 21) were relapsed after transplant. They were all suffering from T-LBL and all had not reached CR before the transplant. The rate of relapse in the cases of T-LBL was 40% (4/10). The overall rate of relapse in the present study was 18.2% (4/22). The median time of relapse was 5 (4–13) months, and these patients died 6–20 months after disease relapse. They all had no aGvHD after transplants. The other two patients died earlier from severe aGvHD (Table 3).

**Figure 1:**
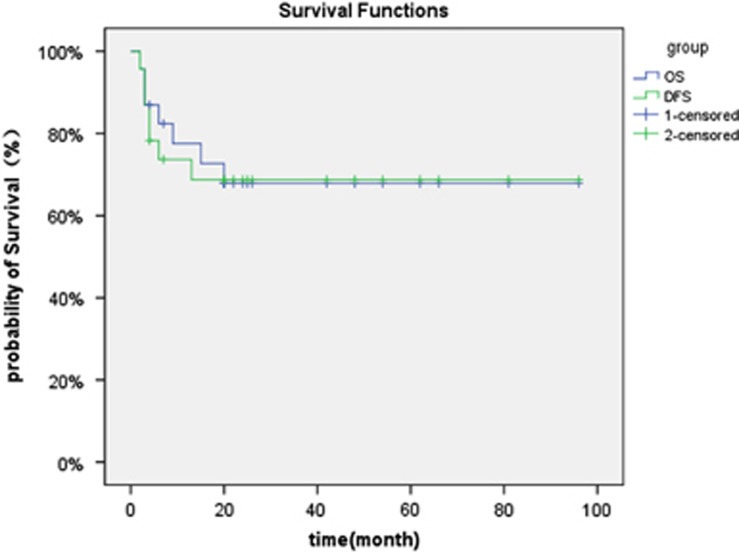
**Probability of overall and disease-free survival.**

**Figure 2:**
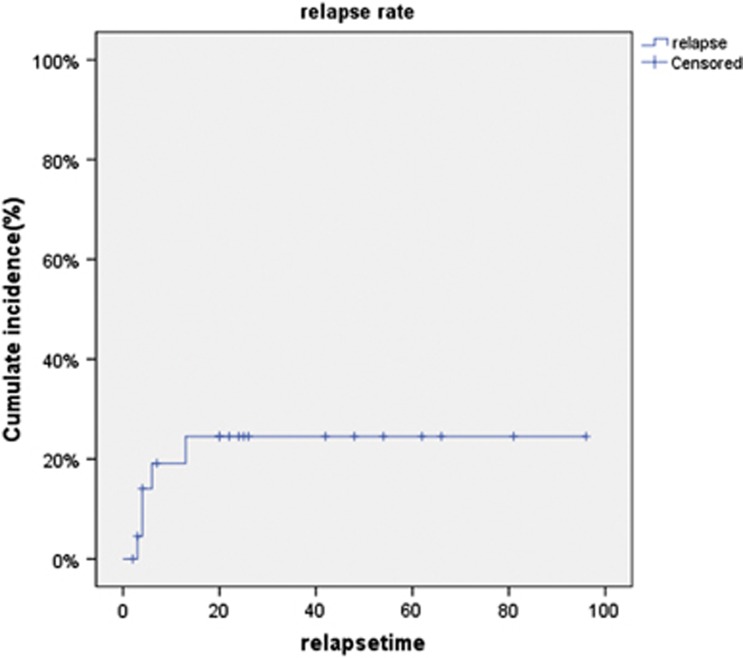
**Probability of relapse.**

At a median follow-up time of 25 months, a median duration of survive was 25 (2–101) month. Sixteen (69.6%) patients are alive, including nine patients in refractory and progressive disease and seven patients in complete or PR before transplant. (Figure 1). One-year estimate of relapse was 21.7% for all patients (Figure 2).

### Graft-versus-host disease

aGvHD grades II–IV occurred in 9 (39.1%) patients and grades III–IV in 3 (13%) patients. Six cases presented with isolated grade II involvement of the skin. The grades III–IV involvement of gun and liver or skin were occurred in the other three patients with HLA-mismatched donor (related or unrelated). All cases received systemic corticosteroids. Two of these (UPN 5,13) required further treatment with anti-CD25 monoclonal antibody basiliximab because of refractory grade IV aGvHD. These two patients died due to transplant-related complications. The cumulative incidence of chronic GvHD (cGvHD) was 30%.

### Regimen-related toxicities and infectious complications

Early toxicities, appearing from day-10 up to+60 after transplant, were graded using the National Cancer Institute—Common Toxicity Criteria 2.0. Eight out of 23 patients developed grade III/IV infections during cytopenia and required i.v. antibiotic or hospitalization. (bacteremia in three patients; bacterial pneumonia in two patients; aspergillus pneumonia in one patient and herpes zoster in two patients). The high levels of CMV DNA copies were detected in 20 patients in the first three months after transplant. And three of them gradually involved into viral hemorrhagic cystitis. All of them were successfully treated with antiviral strategies.

The remaining non-hematological complications within this group of patients were almost exclusively limited to gastrointestinal toxicities. Grade I mucositis was seen in five patients, grade II in eight, grade III in six and grade IV in four. All patients required parenteral nutrition during hospitalization. Eight patients (34.8%) had grade III/IV nausea and another six patients (26.1%) showed grade III/IV diarrhea in the absence of intestinal GvHD. Slight and transient renal and hepatic toxicities occurred in two patients and were limited to grades I and II. One patient showed grade III/IV hepatic toxicity suffering from aGvHD with liver involvement. There was no case of venous occlusive disease. (Table 4 )

## Discussion

ATG is used in allo-HSCT for the prophylaxis of GvHD by *in vivo* T-cell depletion, including the complement-dependent cytotoxic response, antibody-dependent cell-mediated cytotoxicity, the opsonophagocytic role of phagocytic cells and induced apoptosis.^9^ ATG has shown efficacy in preventing aGvHD in allo-HSCT, but its efficacy in cGVHD and long-term outcomes remain controversial. A systematic review and meta-analysis from Du *et al*^10^ reported that prophylactic use of ATG exerted a favorable effect in reducing cGVHD without survival impairment in a long term, although a higher relapse rate is a major threat. But the patients in most reports were involved in myeloid leukemia.^11, 12^ Some scholars also reported the ATG delayed immune reconstitution and hematologic reconstitution and leaded to the increase of the incidence of virus and fungal infections after transplantat.^13^ But these infections are often curable and do not affect the OS and quality of life of the patients.^14^ Because of its strong immune suppression and regulation, ATG as GvHD prophylaxis is generally limited to the unrelated donor, or HLA-mismatched related donor transplantation.

Our preliminary result *in vitro* showed that ATG could inhibit the proliferation of lymphoid tumor cells especially T-cell tumors in a dose-dependent manner.^5^ It was not surprising that T-lymphocytic leukemia/lymphoma cells displayed high sensitivity to ATG because T-lymphocytes were predominant in thymic tissues. These results are consistent with those of previously published reports.^15, 16^ Therefore, ATG may be used as anti-lymphocyte tumor biotherapeutics as anti-CD20 monoclonal antibody rituximab in treatment of B-cell lymphomas to increase the role of chemoradiotherapy in the conditioning regimen. Therefore, all cases enrolled in this study were T-cell tumors, including T-ALL, T-lymphocyte lymphomas. We proposed that addition of ATG to conditioning regimen could reduce the rate of the T tumor recurrence and help to remove the residual tumor lesions after transplant. Our primary purpose was to evaluate practicability, safety and complication profile of this regimen as a myeloablative conditioning therapy. Our secondary goal was to evaluate the long-term anti-leukemic effect of this treatment.

We reported here on the 23 patients with aggressive T-cell tumors who received allogeneic matched or mismatched related or unrelated donor HSCT after myeloablative conditioning regimen containing ATG (10 mg/kg). Twenty-two (95.7%) patients achieved a CR after the conditioning therapy, including 13/14 (92.9%) patients failed to reach a CR or PR at the time of transplant. With a median follow-up of 25 months, 16 (69.6%) cases were alive and free from disease. This result is better than the results reported by other groups,^17, 18, 19, 20, 21^ especially in case of the unfavorable state of diseases.

With 34.8% patients suffering from grade III/IV infectious complications during aplasia, infections were the main risks for our patients within the early phase post transplant. But the infections was nonfatal and resolved in all patients. Gastrointestinal toxicities like nausea/vomiting, mucositis and diarrhea evoked by this regimen were the most common side effects. Grades III–IV nausea/vomiting was seen in 34.8%, grades III–IV mucositis in 43.5% and grades III–IV diarrhea in 26.1%, which was not higher than the studies with other myeloablative conditioning regimens.^22, 23^ Recently Some scholars^10, 13^had also reported that high levels of ATG antibodies to Ag(s) expressed on T- and B cells were associated with a low risk of aGvHD and a high risk of viral but not bacterial or fungal infections. The common viral infection were CMV viremia and BK virus cystitis. But these infections were often curable and do not affect the OS and quality of life of the patients. In the present study, the rate of cytomegalovirus infection was high. But there was also no patient dying from cytomegalovirus infection or the other infectious complications.

The thymoglobulin doses of 4–6 mg/kg is commonly used as GvHD prophylaxis in the unrelated donor, or HLA-mismatched related donor transplantation but not recommended in the HLA-matched related donor transplantation.^14, 24^ In order to observe the anti-tumor effect of this conditioning regimen in the aggressive T-cell tumor patients in CR, PR and relapse or progressive disease, thymoglobulin dose in the conditioning regimen were increased here to total dose of 10 mg/kg even in the HLA-matched related donor transplantation. The purpose is to reduce primary disease recurrence, improve rate of CR, DFS and OS after transplantation, as well as reduce the incidence and severity of GvHD. With 9 out of 23 patients developing an aGvHD (39.1%) and 6 out of 20 patients showing cGvHD (30%), the incidences of aGvHD and cGvHD after allogeneic peripheral blood stem cell transplantation in the present study were comparable to the previous studies.^25^ aGvHD was observed in four cases receiving graft from MUD and three cases receiving graft from haplogeneic donors. Therefore, these results indicated that the ATG-based regimen was sufficiently practicable and comparable with other myeloablative conditioning regimens with regards to TRM, although the rates of gastrointestinal toxicity and cytomegalovirus infection were higher.

Although this study did not have sufficient power to analyze outcome by individual histologies, we were interested in determining whether there was any difference in outcome between the PTCL and the T-ALL/LBL.

In the present study, the aggressive peripheral T-cell lymphoma consisted of hepatosplenic T-cell lymphoma, γ/δT-cell lymphoma as well as PTCLs not otherwise specified. All patients in PD received several kinds of chemotherapy before allo-HSCT and were generally considered not to be cured with conventional approaches. These seven patients with PTCL obtained CR after the conditioning regimen. The CR rate was much higher than 56% reported by Le Gouill *et al.*^17^ They remained alive without evidence of disease. The median duration of DFS reached to 47 (12–86) months. The longest survivor was a female patient who relapsed post autologous HSCT. This result compares well with other conditioning regimens that have been used in the past, especially considering the unfavorable status of diseases in our patients.^19, 20, 21, 26^

There is a significant biological and clinical overlap between neoplasms diagnosed as LBL and ALL. Accordingly, LBL and ALL were considered the same disease with characteristics, immunophenotype and response to chemotherapy for decades.^27^ In those relapsed LBL patients who have a particularly poor outcome, conventional salvage chemotherapy were not satisfactory.^28, 29, 30, 31, 32^ Here,we observed 16 patients with T-ALL/LBL, including five cases of high-risk T-ALL with initial WBC>=100 000/ml and 11 cases of T-LBL in stages IV diseases. Ten patients could not achieve CR with the conventional induction or re-induction therapy at the time of transplantation. With a median follow-up of 25 Months, six cases of CR before transplant remained alive and free of diseases, and a median duration of DFS was 6 (–31) months in nine patients with refractory T-ALL/LBL who obtained CR after transplant. Four cases relapsed on month 4, 4, 6 and 13, respectively. It is suggested that allo-HSCT is one of choice for post-remission treatment in the highly aggressive T-cell tumors. How to prevent relapse post transplant remains to investigate.

## Figures and Tables

**Table 1:** Patient demographics

	*Median (range)*
Age in years	26 (7–55)
*Sex*	*No. (%)*
Male	13 (56)
Female	10 (44)
	
*Diagnosis*	*No.*
T-ALL	5
WBC〉 100 × 10^9^/l	5
T-cell lymphoma	18
Angioimmunoblastic T-cell	1
Peripheral T-cell NOS	3
Hepatosplenic T-cell lymphoma	2
γ/δ T-cell lymphoma	1
T-lymphoblastoid cell lymphoma	11
*Status of lymphoma*	
Stages III–IV or bulky	18
Mediastinal mass	
None	9
<10 cm	4
>10 cm	5
Bone marrow involvement	10
Pleural and/or pericardial effusion	6
B symptoms	12
	
*Prior autologous transplantation*	*No. (%)*
Yes	2 (8.7)
No	21 (91.3)
Courses number of prior therapies	8 (2–18)

*Disease status at transplantation*	
CR1	4
CR2	2
PR	3
NR/PD	14
*Donor characteristic*	
Matched unrelated	5
Mismatched unrelated (8/10)	5
Matched related	5
Mismatched related	8

Abbreviations: CR1, first complete remission; CR2, second complete remission; NOS, not otherwise specified; NR, no response; PD, progressive disease; PR, partial remission.

B symptoms, tumor fever higher than 38°C, night sweats and/or weight loss >10%. Bulky is a mass >10 cm in size.

**Table 2:** Disease status before and after HSCT

	*n*	*Before HSCT*			*After HSCT*			*Survival*
		*CR*	*PR*	*NR/PD*	*CR*	*PR*	*NR/PD*	
T-ALL	5	3		2	5			4
T-LBL	11	3	3	5	10	1		5
PTCL	6			6	6			6
AITL	1			1	1			1
Total	23	6	3	14	22	1		16 (69.6%)

Abbreviations: AITL, angioimmunoblastic T cell lymphoma; ALL, acute lymphoblast leukemia; CR, complete remission; PR, complete remission; PD, progressive disease; PTCL, peripheral T-cell lymphoma.

**Table 3:** Outcome of treated patients

*UPN*	*Diagnosis*	*Donor (mismatched)*	*Status at transplant*	*Engraftment*	*Acute GvHD*	*Chronic GvHD*	*Current status (month)*
1	T-lymphoblastoid cell lymphoma	MSD (−)	CR1	Yes	—	—	Alive CR +101
2	Peripheral T-cell lymphoma	MSD (−)	Refractory relapse	Yes	II	Extensive	Alive CR +86
3	T-ALL	MUD 8/10	CR1	Yes	III–IV	Limit	Alive CR +71
4	hepatosplenic T-cell lymphoma	MUD (−)	Primary refractory	Yes	II	—	Alive CR +67
5	T-ALL	MUD 8/10	Relapse	Yes	IV		Dead TRM +3
6	γ/δ T-cell lymphoma	MSD (−)	Primary refractory	Yes	II	Limit	Alive CR +53
7	T-lymphoblastoid cell lymphoma	MUD (−)	PR	Yes	—	—	Dead relapse 4(20)
8	T-lymphoblastoid cell lymphoma/leukemia	MUD (−)	CR1	Yes		—	Alive CR +59
9	T-ALL	MSD 8/10	CR2	Yes	II	Limit	Alive CR +30
10	T-lymphoblastoid cell lymphoma/leukemia	MUD 8/10	PR	Yes	—	—	Dead relapse +13(15)
11	Angioimmunoblastic T-cell lymphoma	MUD 8/10	Primary refractory	Yes	—	—	Alive CR +47
12	T-lymphoblastoid cell lymphoma	MUD 8/10	PR	Yes	—	Limit	Alive CR +31
13	T-lymphoblastoid cell lymphoma	MSD 3/6	PD	Yes	IV	—	Dead TRM +2
14	T-lymphoblastoid cell lymphoma	MSD 3/6	PD	Yes	—	—	Dead PD +3
15	T-lymphoblastoid cell lymphoma	MSD 3/6	PD	Yes	—	—	Dead relapse +4(6)
16	Peripheral T-cell lymphoma	MSD (−)	PD	Yes	—	—	Alive CR +29
17	Peripheral T-cell lymphoma	MSD (−)	PD	Yes	—	—	Alive CR +25
18	T-ALL	MSD 3/6	CR2	Yes	I	—	Alive CR +27
19	T-ALL	MUD (−)	Relapse	Yes	II	—	Alive CR +25
20	T-lymphoblastoid cell lymphoma	MSD 3/6	PD	Yes	II	Limit	Alive CR +25
21	T-lymphoblastoid cell lymphoma	MSD 3/6	Relapse	Yes	—	—	Dead Relapse +6 (9)
22	Hepatosplenic T-cell lymphoma	MUD (−)	PD	Yes	—	—	Alive CR +12
23	T-lymphoblastoid cell lymphoma	MSD 3/6	CR1	Yes	I	—	Alive CR +9

Abbreviations: ALL, acute lymphoblast leukemia; CR, complete remission; GvHD, graft-versus-host disease; MSD, matched sibling donor; MUD, matched unrelated donor; PD, progressive disease; TRM, treatment-related mortality; UPN, unique patient number.

**Table 4:** Non-hematological toxicity

*CTC 2.0 grade*	*0*	*I/II*	*III/IV*
Infection (*n*) (%)	15 (65.2)	0	8 (34.8)
Nausea/vomiting (*n*)(%)	0	15 (65.2)	8 (34.8)
Mucositis (*n*) (%)	0	13 (56.5)	10 (43.5)
Increase in transaminases/bilirubin (*n*) (%)	0	1 (4.3%)	1 (4.3%)
Increase in creatinine (*n*) (%)	0	1 (4.3%)	0
Diarrhea (*n*) (%)^a^	0	17 (73.9)	6 (26.1)

Abbreviation: GvHD, graft-versus-host disease.

a Not associated with GvHD.
